# Malignant melanoma of the oral cavity. Review of the literature and experience in a Peruvian Population

**DOI:** 10.4317/medoral.17477

**Published:** 2011-12-06

**Authors:** Janet O. Guevara-Canales, Mario M. Gutiérrez-Morales, Sonia J. Sacsaquispe-Contreras, Juvenal Sánchez-Lihón, Rafael Morales-Vadillo

**Affiliations:** 1 Faculty of Dentistry. “San Martín de Porres” University, Lima, Peru; 2 Department of Medicine, Surgery and Oral Pathology. Faculty of Dentistry. Peruvian university “Cayetano Heredia”, Lima, Peru; 3 National Institute for Neoplastic Diseases “Dr. Eduardo Cáceres Graziani” (INEN), Lima, Peru

## Abstract

Objective: To determine the epidemiological profile of malignant melanoma cases treated at the National Institute for Neoplastic Diseases “Dr. Eduardo Caceres Graziani” (INEN) over the period 1952 to 2008. Study Design: All clinical records with complete data of patients presenting a histopathological diagnosis of malignant melanoma of the oral cavity were reviewed. Data such as age, gender, location, tumor size, disease length, presence of metastasis, treatment received and year of admission were recorded. Results: During the study period 97 cases were found. The average age of patients was 52.85±1.6 years old mostly between 50 and 59 years old; the predominant gender was the female. The most common location was the palate and there was 58.8% of cases with a tumor size bigger than or equal to 4 cm. The length of the disease in 38.1% of the cases was longer than a year and in great part of the cases (69.1%) there was no metastasis. The treatment of choice was the surgery plus radiotherapy in 38.1% of the cases. According to the admission date it was also noted that the number of cases is increasing. Conclusion: The results of this study demonstrate a late diagnosis and an increasing frequency of this neoplasia in the oral cavity.

** Key words:** Melanoma, oral cavity, epidemiology.

## Introduction

The malignant melanoma of the oral cavity is a neoplasia developed from melanocytic cells that are in the basal layer of the mucosa (1), its incidence is of 1.2 cases per 10 million inhabitants per year (2), with a variation between 0.2% to 8% of all the melanomas (3,4) and 0.5% of all the malignant neoplasias of the oral cavity (5).

Its etiology is unknown, although sometimes it is placed on pre-existing long-term melanosis involving 33 to 55% of the mucosal melanomas of the head and neck (6), other possible etiological factors for this neoplasia are: mechanical trauma such as denture irritation (7), use of tobacco, exposure to formaldehyde (8) and alcohol. 

Most cases occur between the fourth and the seventh decade of life, with an average of 55-57 years old (9), not very frequently below 30 years old (10). Apparently the malignant melanoma of the oral cavity has a predilection for the male gender (8), in a male-female ratio of 2:1 (11,12). The areas in which they appear in order of frequency are: the hard palate (where 40% of the cases have been reported) (2), followed by upper gingival mucosa (13), lower gingival mucosa, buccal mucosa, tongue and floor of mouth (14). The clinical characteristics are variable, such as macular lesions, plate (with horizontal growth, which often corre-spond to melanomas in situ in the histopathologic exam) and nodular (with clinical ulceration, usually of an invasive type or combined at a microscopic level) (15); their colors vary from dark blue to black and their edges are regular or irregular. The symptoms of the oral mucosal melanoma include: bleeding (referred to in the diagnosis as the most frequent sign) (10), pain (it often appears late) and presence of melanotic pigmentation (in one third of the patients before the diagnosis) (16). 

Unlike the cutaneous melanoma in the oral mucosa, there is no well-defined clinical and pathological classification (14), that is, Clark’s criteria for the invasion level and the prognosis of the cutaneous melanoma are not applicable to oral melanomas due to the lack of histological points of reference similar to the papillary and reticular dermis (17), nevertheless, some studies have com-pared oral melanomas with the acral lentiginous melanoma and with the cutaneous nodular melanoma (2). Most authors use the classification of the Western Society of Teachers of Oral Pathology (WESTOP), which divides them into a relatively simple sys-tem according to its histopathological pattern in: (a) melanoma in situ, delimited to the epidermis and its junction with the connective tissue; (b) invasive melanomas, in which the neoplasia extends into the connective tissue and (c) melanomas with a combined pattern between invasive and in situ (18,19).

Histologically it is characterized by the proliferation of atypical melanocytes with a wide variety of shapes, including the one of the spindle, of plasmacytoid cells, clear cells and some epithelioid cells (18), located along the junction between the epithelial and the connective tissue, as well as invading the connective tissue (2). It also describes histological stages of the oral malignant melanoma in 3 phases: Stage I primary site, Stage II with lymph node metastasis and Stage III with distant metastasis (20). The immunohistochemistry has referred positivity in a varying level for the antigens related to the melanoma: NKI/C-3, S-100 protein, gp100 (HMB-45), Mart-1 (Melan-A) (21), vimentin, tyrosinase and microphthalmia transcription factor (MiTF) that are useful in the diagnosis. The vimentin is the most consistent, but the less useful for the diagnosis; the S-100 protein positivity is nonspecific, but due to the fact that it is negative in most of the tumors that are considered in the differential diagnosis, this stain is very important; the HMB-45 is a much more specific marker than protein S-100; the Melan-A is positive in approximately 80% of melanomas and MiTF positivity is above the 90% (22). The oral melanoma has a metastatic predilection for lymphonodes (18), lungs, liver, brain and bones.

The treatment of choice of the oral melanomas consists of the complete surgical resection of the lesion with safety margins, addi-tionally, radiotherapy and chemotherapy can be used (23,24).

It is assumed that the worst prognosis of oral mucosal melanoma with regard to the cutaneous melanoma is due to a late diagnosis, to differences in its histopathological behavior, to the increased trend to deep invasion, to the early hematogenous metastases, to the anatomical peculiarities of the region with difficulty in the surgical resection with disease-free margins (25) and to the absence of standardized treatment protocols (14). The prognosis of this lesion is poor with a survival rate at 5 years for patients with melanoma of the oral cavity within a range of 15-38% after the diagnosis (26). 

Regarding the melanoma of the oral cavity, most of the available information on its clinical and epidemiological features comes from a series of small cases, so that the knowledge of the features of this pathology in larger populations are of great meaning for the knowledge of the natural history of this lesion.

## Material and Methods

The present study is descriptive, retrospective, transversal and of a series of cases. The study sample corresponds to patients with clinical records that had an anatomopathological diagnosis of melanoma of the oral cavity treated in the National Institute for Neoplastic Diseases “Dr. Eduardo Cáceres Graziani” (INEN) over the period 1952 to 2008. The information was collected in data collecting forms especially prepared for this study, for the database Microsoft Excel program was used for its further statistical analysis in Statistical Package for Social Sciences (SPSS) version 15.0. Tables of frequencies were made for the descriptive analysis of each variable, the percentages were indicated.

## Results

This review included 97 cases of oral melanoma treated in the National Institute for Neoplastic Diseases “Dr. Eduardo Cáceres Graziani” (INEN) over the period 1952 to 2008. The average age group of this population group was found in 52.85±1.6 years, in a range between 13 and 96 years old, most cases are observed between 50 and 59 years old (26.8%). The greatest frequency of patients with oral melanoma is in the female gender with a 52.6%, the male:female ratio was 0.9:1. The greatest frequency of location was in the palate in 47.4% of cases, followed by the upper alveolar ridge with 27.8%, the lowest percentage regarding site was found on the floor of the mouth in only 1.0%. With regard to the tumor

**Figure 1:**
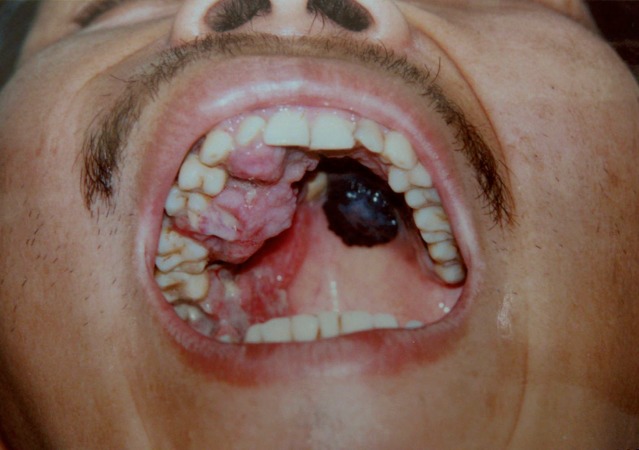
Clinical setting of a 31-year-old patient with oral melanoma in hard palate with a 7-year disease length.

**Figure 2:**
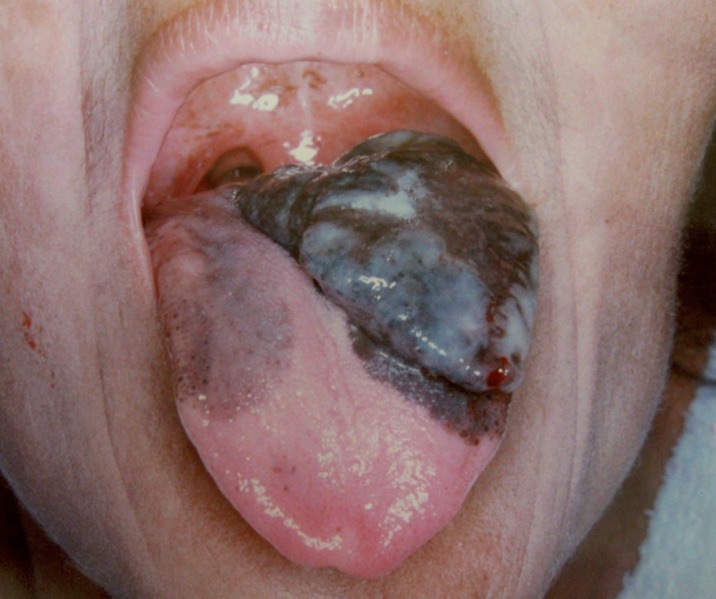
Female 44-year-old patient with oral melanoma in tongue with a 2-year disease length.

size, an average of 4.26 cm was found in a range of 0.5 to 10 cm, the great part of patients 58.8% present tumors equal to more than 4 cm. (Figs. 1 and 2). The 38.1% of the cases had disease length of more than 1 year, followed by patients with less than 3 months (30.9%). The type of treatment generally used was the surgical complemented with radiotherapy (38.1%), followed by surgical treatment alone (21.6%). Most patients did not present metastasis (69.1%), representing more than two thirds of the population studied. The percentage of cases

**Table 1:**
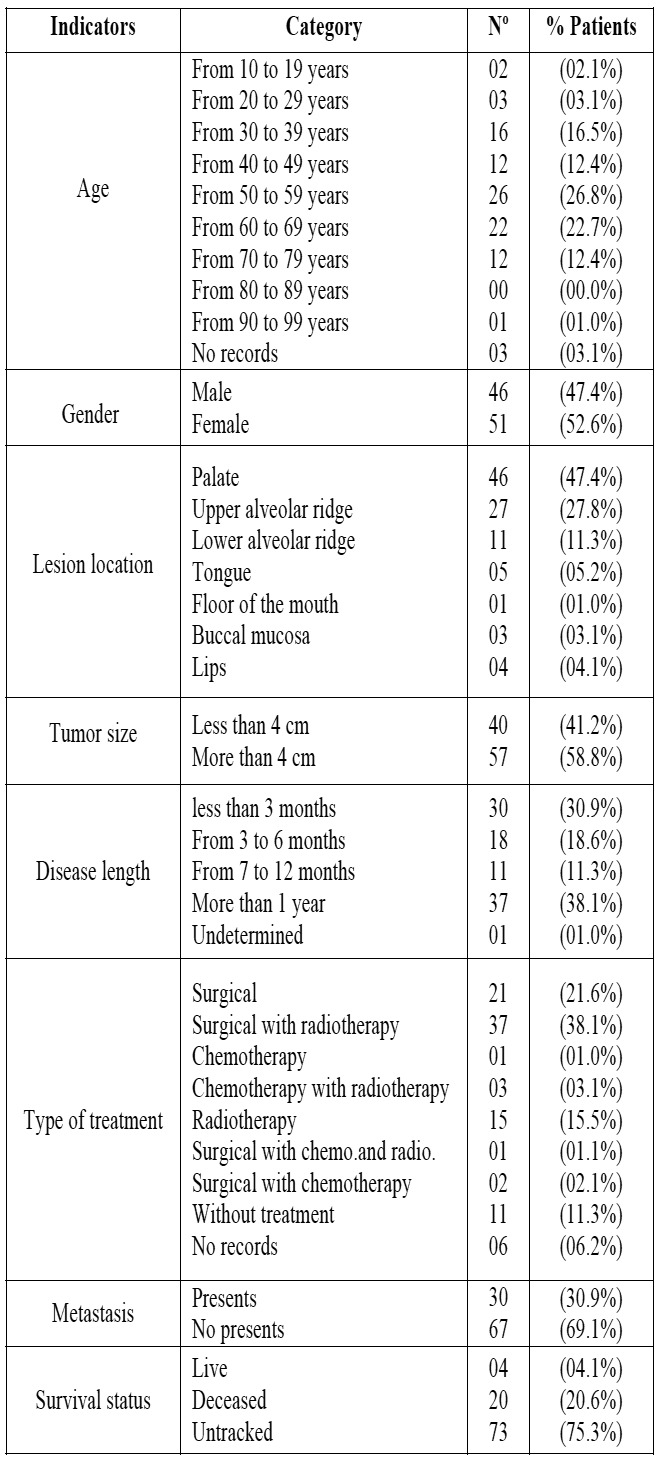
Distribution of study indicators according to categories in patients with oral melanoma of the National Institute for Neoplastic Diseases “Dr. Eduardo Caceres Graziani”, Lima, Peru, over the period 1952-2008.

**Figure 3:**
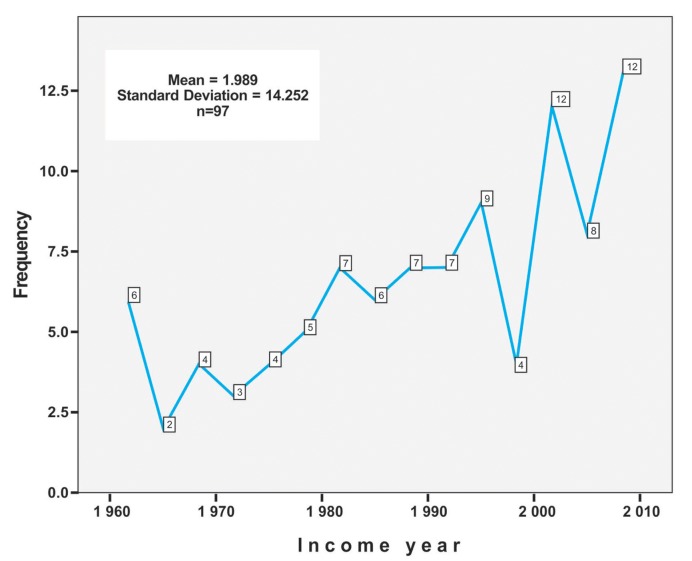
Number of cases of oral melanoma as per year. National Institute for Neoplastic Diseases "Dr. Eduardo Caceres Graziani, Lima, Peru (1952-2008).

lost sight of was 75.3%, of patients who died of 20.6% and 4.1% patients alive, therefore could not perform the respective statistical analysis of survival. ( Table 1). It was found that there is a trend to the increase of the number of cases as the time goes by, a greater percentage of cases was observed in 2008 (6.8%), followed by the year 2007 (6.2%) (Fig. 3).

## Discussion

The melanoma of the oral cavity is not so frequent malignant neoplasia and it accounts for 0.2 to 8% of all the melanomas affecting the organism (3). It presents a poor prognosis due to several characteristics such as: remaining asymptomatic for a long time, which justifies its late discovery (14). The edges do not have the typical induration of the carcinomas resulting difficult to be recognized as malignant and also the rich vasculature of the area contributes to its spreading.

In the present study 97 cases of oral melanoma treated in the National Institute for Neoplastic Diseases “Dr. Eduardo Cáceres Graziani” (INEN) over a period of 56 years (1952-2008) are reported and described. This is an important and significant sample since it took place in an institution specialized in malignant neoplasias, it is a referential center at national level. 

The average age of the cases studied was 52.85 years old, which agrees with Chidzonga’s et al. study (24) in which the average age was 56 years old, similar to Lopez-Graniel et al. study (15) where the sixth decade of life is the average of patients with oral melanoma. 

Doval et al. (27) and Tanaka et al. (28) state that the oral melanoma is more frequent in people older than 40 years old, agreeing with this study, in which 75% of the cases were older than 40 years old, while the highest prevalence in the range of 50-59 years old (26.8%), agreeing with Rapidis et al. (10) who mentions that this disease is rare before the age of 30.

This study determined that there is a slight female predominance (52.6%), as well as in the results of Meleti’s et al. study (21), however, the review of the literature finds a male predominance.

It was noted that the location of the most frequent lesion was in the palate (47.4%), followed by the upper maxillary mucosa (27.8%), finding relation with that reported by Meleti et al. (29) who describes 41 of 119 cases with lesion in the palate (34.4%), followed by the upper maxillary mucosa; as in Doval’s et al. research (27) who points out that from 14 cases, 11 had a lesion in the palate. 

Regarding the tumor size of the lesion the bigger and equal to 4 cm was frequent in 57 patients (58.8%), this result is related to Lopez-Graniel et al. study (15) in which 66.6% of cases had a size of 4 cm or more.

Most cases presented a disease length longer than 1 year (38.1%), the patients referred that they did not feel pain, some of them mentioned that they had a mark for several years and as the time went by it increased in size until they felt discomfort, was ob-served that the cases occurred more frequently after 6 months the disease started (68.75%). 

A complete surgical excision with radiotherapy was the type of treatment of choice (38.1% of cases), coinciding with Doval’s et al. study (27).

Tanaka et al. (28) mentioned that of 35 cases examined, 18 had metastasis (52.43%), in this studied population, only a third part of the cases developed metastasis (30.9%).

Lopez-Graniel et al. (15) reports that only 6.6% of all the cases studied survived more than 5 years, although, the survival time in this study could not be performed since the information of the clinical records was incomplete.

In conclusion, over the years a continuous increase of melanoma cases in the oral cavity was observed within the period studied presenting higher prevalence in the sixth decade of life and being most often located in the palate. Moreover, it was found that at the moment of the diagnosis the tumor size was bigger than or equal to 4 cm. and the excisional surgery complemented with ra-diotherapy was the treatment most used in this population group. Since many medical records had no updated follow-up records of patients the survival was not possible to be determined. The results of this study suggest that an early diagnosis is required to improve the prognosis of patients.
